# Rescue and characterization of the first West African Marburg virus 2021 from Guinea

**DOI:** 10.1016/j.heliyon.2023.e19613

**Published:** 2023-08-29

**Authors:** Isabel von Creytz, Gesche K. Gerresheim, Clemens Lier, Jana Schneider, Martin Schauflinger, Marcel Benz, Lennart Kämper, Cornelius Rohde, Markus Eickmann, Nadine Biedenkopf

**Affiliations:** a Institute of Virology, Philipps-University Marburg, 35043 Marburg, Germany; b German Center for Infection Research (DZIF), Partner Site Giessen-Marburg-Langen, 35043 Marburg, Germany

**Keywords:** Marburg virus, Filovirus, Reverse genetics, Full-length clone, Virus replication, Remdesivir

## Abstract

Marburg virus (MARV) is a causative agent of a severe hemorrhagic fever with high fatality rates endemic in central Africa. Current outbreaks of MARV in Equatorial Guinea and Tanzania underline the relevance of MARV as a public health emergency pathogen. In 2021, the first known human MARV case was confirmed in Guinea, West Africa. Since no infectious virus could be isolated from that fatal case in 2021, we generated recombinant (rec) MARV Guinea by reverse genetics in order to study and characterize this new MARV, which occurred in West Africa for the first time, in terms of its growth properties, detection by antibodies, and therapeutic potential compared to known MARV strains. Our results showed a solid viral replication of recMARV Guinea in human, bat, and monkey cell lines in comparison to other known MARV strains. We further demonstrated that replication of recMARV Guinea in cells can be inhibited by the nucleoside analogue remdesivir. Taken together, we could successfully reconstitute *de novo* the first West African MARV from Guinea showing similar replication kinetics in cells compared to other central African MARV strains. Our reverse genetics approach has proven successful in characterizing emerging viruses, especially when virus isolates are missing and viral genome sequences are incomplete.

## 1 Introduction

Marburg virus (MARV, species *Orthomarburgvirus marburgense*) belongs to the family of *Filoviridae (*order *Mononegavirales)*, together with the closely related Ebola virus (EBOV, species *Orthoebolavirus zairense*). MARV causes a severe hemorrhagic fever (MVD, Marburg virus disease) with high fatality rates in humans [[1], [2], [3]]. The natural reservoir of MARV are fruit bats of the species *Rousettus aegyptiacus* (Egyptian fruit bat), whose wide geographic distribution presents an increased potential for MARV zoonotic transmission in large areas of sub-Saharan and West Africa [[4], [5], [6], [7]]. While there has been considerable progress in preclinical and clinical MARV vaccine development in the last years, an approved MARV-specific vaccine is still lacking [8,9]. MARV has been therefore emphasized by the World Health Organization (WHO) as one of the pathogens most likely to cause public health emergencies [10].

MARV was first identified in 1967 when lab workers in Marburg and Frankfurt (Germany), as well as in Belgrade (Serbia) became infected after handling African green monkeys imported from Uganda [3,11,12]. Since then, sporadic MARV outbreaks have been reported in Kenya, Democratic Republic of the Congo, Angola and Uganda with the so far largest outbreak in 2004–2005 in Angola (252 cases and a fatality rate of 90%) [13]. In 2021, a fatal MARV case was reported in Guinea, West Africa in the Gueckedou prefecture [14]. This was the first time, MARV was detected in West Africa and raised concerns about a possible epidemic in the region, where the so far largest EBOV outbreak started in 2014 [15,16]. Fortunately, no one of the 173 contacts, including 14 high-risk contacts, has become infected with MARV Guinea [17]. Sequencing of the virus from the only infected patient showed that the closest related MARV strains are MARV Angola and a fruit bat isolate from Sierra Leone [14]. Since then, the relevance of MARV as a causative public health emergency pathogen has increased considerably with three fatal MARV cases in Ghana in 2022 and the recent MARV outbreaks in Equatorial Guinea (February to June 2023) and in Tanzania (March to June 2023) with 35 and 6 fatal cases, respectively [[18], [19], [20]].

In order to characterize this first West African MARV 2021 from Guinea in comparison to other MARV strains originated from central Africa, we started a reverse genetic approach to generate infectious recombinant (rec) MARV Guinea due to the absence of a natural isolate. Given the potential of MARV to cause outbreaks with high fatality rates in humans, we found it of great importance to generate and characterize this newly emerged Marburg virus, which was first described in West Africa. Since the sequence of MARV Guinea was incomplete at both genome ends, we filled the missing nucleotides (nts) with a closely related MARV strain Angola sequence (GenBank accession number DQ447654). We could validate the functionality of these sequences and of the viral nucleocapsid proteins NP, VP35, VP30, and L of MARV Guinea in a MARV Guinea-specific minigenome reporter assay. Using *de novo* gene synthesis and subcloning of cDNA cassettes [21], we generated full-length MARV Guinea cDNA under the control of a T7 promotor and could successfully rescue infectious recMARV Guinea with the help of additionally transfected viral nucleocapsid proteins and a T7 polymerase. We next characterized viral replication of recMARV Guinea in relevant human, bat and monkey cell lines in comparison to other MARV strains (recMARV Musoke, natural isolates of MARV Musoke and Leiden). Our results showed a solid viral replication of recMARV Guinea and a pronounced cytopathic effect (CPE) in human and monkey cell lines. Interestingly, we observed enhanced viral replication of recMARV Guinea in bat cells, when compared with recMARV Musoke. We further demonstrate that replication of recMARV Guinea in cells can be inhibited by the nucleoside analogue remdesivir (GS-5734). Taken together, we could successfully reconstitute *de novo* the first West African MARV from Guinea that shows similar characteristics in cells compared to other MARV strains. Our reverse genetics approach further demonstrates the possibility to characterize emerging viruses of concern, especially when virus isolates are missing and/or virus sequences are incomplete.

## 2 Materials and methods

### 2.1 Cells

HuH7 (human hepatoma cells, fully matching the STR reference profile of cell line HuH7), HEK293F (primary embryonic human kidney, fully matching the STR reference profile of cell line HEK293), Vero C1008 (clone E6, Vero E6, ATCC CRL-1586) (African green monkey kidney cells) and RoNi/7.1 (*Rousettus aegyptiacus* kidney cells kindly provided by Marcel Müller, Charité Berlin) [22] cells were cultivated in Dulbecco's modified Eagle medium (DMEM) supplemented with fetal bovine serum (10% for maintenance and 3% for experiments), penicillin (50 units/ml) and streptomycin (50 mg/ml) (10% resp. 3% DMEM ++) at 37 °C and 5% CO_2_.

### 2.2 Viruses

All experiments with MARV were performed at the BSL-4 facility of the Philipps-University Marburg according to national regulations. MARV strain Musoke (GenBank accession number DQ217792) and Leiden (GenBank accession number JN408064) stocks were produced in Vero E6 cells infected with a multiplicity of infection (MOI) of 0.1 followed by an incubation for 7 days at 37 °C. Viral titers (plaque forming units, PFU) were calculated based on immunoplaque assay. Therefore, confluent Vero E6 cells were infected in a 24 well plate with different dilutions of the respective virus. The virus-containing inoculum was replaced by 500 μl 2% carboxymethylcellulose (CMC) in Minimum Essential Medium (MEM, containing penicillin (50 units/ml) and streptomycin (50 mg/ml)) and cells were incubated at 37 °C for 4 days. The cells were then fixed with 4% paraformaldehyde (PFA)/DMEM for 48 h, discharged from the BSL-4 laboratory and prepared for intracellular immunofluorescence staining. Immunofluorescence staining was performed using a goat α-MARV serum (dilution 1:2000; [23]) and a donkey α-goat IgG (H + L) Cross-Adsorbed Alexa Fluor™ 488 secondary antibody (dilution 1:400). Plaques were counted on a fluorescence microscope. To calculate the titer, the number of plaques was multiplied by the dilution factor and expressed as PFU/ml.

### 2.3 Cloning of Marburg virus Guinea plasmids

Since the obtained sequence from the original patient (GenBank accession number OK665848) was not complete [14], we substituted the missing nucleotides (nts) with sequences of a closely related MARV Angola strain (GenBank accession number DQ447654). The substituted *leader* sequence was generally highly conserved. In total, we substituted the first 119 nts (*leader* sequence and the first 16 nts of the *NP* gene), the last 10 nts of the genome (*trailer* region), and additionally 13 missing internal nucleotides (N2691T, N2693A, N2815T, N3249G, N5392T, N6056G, N6626T, N6629A, N7278C, N10953G, N12624C, N12846T, N17450C).

We first cloned MARV Guinea-specific plasmids of all viral proteins NP, VP35, VP40, GP, VP30, HA-tagged VP30, VP24, and L each in pCAGGS eukaryotic expression vectors.

MARV Leiden plasmids (L, NP, GP, VP40, VP35, and VP30 in pCAGGS vector) were similarly cloned. Plasmids encoding viral proteins of MARV Musoke were described elsewhere [24,25]. In order to verify the functionality of the resubstituted genome ends, we cloned a monocistronic MARV Guinea minigenome plasmid encoding a *Renilla* luciferase as reporter gene.

To generate a full-length (FL) plasmid encoding MARV Guinea, we used an approach based on *de novo* gene syntheses with the substitution of missing nts by MARV strain Angola (GenBank accession number DQ447654, see above). Additionally, we mutated two nts in the GP coding region to generate a *Sac*II restriction enzyme site (A7522C and A7525G, both silent mutations) for both cloning purposes and as unique identification as a recombinant virus. Sequences were cloned into three cassettes and finally ligated into one complete FL plasmid under the control of a T7 promotor [21].

Detailed cloning strategies and primers are available on request.

### 2.4 Marburg virus-specific minigenome reporter assay

Marburg virus-specific minigenome reporter gene assays were performed in either HEK293F (8x10^5^ cells/6 well), or HuH7 cells (2x10^5^ cells/6 well). Cells were transfected with plasmids encoding the viral nucleocapsid proteins essential for viral transcription and replication together with a T7-driven MARV-specific minigenome encoding a *Renilla* luciferase [24,25]. Plasmids were transfected using the following DNA concentrations: 500 ng NP, 100 ng VP35 , 50 ng MARV Guinea VP30 or 100 ng MARV Musoke VP30, 2 μg MARV Guinea L or 1.5 μg MARV Musoke L, 2 μg MARV Guinea-specific minigenome or 0.5 μg MARV Musoke-specific minigenome, and 100 ng T7-polymerase. For normalization, a plasmid encoding a Firefly luciferase, pGL4 vector, was additionally transfected (25 ng). Transfections were performed using *Trans*IT LT-1 (Mirus) according to the manufacturer's protocol. Cells were incubated at 37 °C for 48 h. Cells were then washed once with phosphate-buffered saline (PBS_def_), harvested in fresh PBS_def_ and lysed in 100 μl 1x Passive Lysis buffer at room temperature (RT) for 15 min. 5 μl cell lysate each was used (for HEK293F cells 1:100 dilution) to measure *Renilla* luciferase (MARV-specific reporter gene activity) or Firefly luciferase activity with *Renilla*-Juice or Beetle-Juice BIG KITs (both PJK), respectively in a CentroLB 960 luminometer (Berthold Technologies). *Renilla* luciferase raw data were normalized with the corresponding Firefly luciferase raw data.

### 2.5 Rescue of recombinant Marburg virus Guinea

A mixed culture of HuH7 cells and Vero E6 cells (each cell line: 1x10^5^ cells/6 well, passage 0) was transfected using plasmids encoding the FL MARV Guinea genome (2 μg), T7 polymerase (0.5 μg) and helper plasmids coding for the MARV polymerase L (2 μg), NP (0.5 μg), VP35 (0.1 μg) and VP30 (0.1 μg) of either MARV strain Guinea, Musoke, or Leiden. As a positive control, we used the FL plasmid of MARV Musoke. Transfections were performed in technical duplicates using *Trans*IT LT-1 (Mirus) according to the manufacturer's protocol. 4 h post transfection (hpt) a medium change was performed to remove residual transfection reagent. Supernatants were transferred 7 days post transfection onto fresh Vero E6 or HuH7 cells (passage 1, p1) and again 7 days later (passage 2). Development of cytopathic effect (CPE) was monitored and the supernatants were analyzed for the presence of VP40 by Western blot analyses (see 2.8), as well as for viral RNA. Viral RNA was extracted using the QIAamp Viral RNA Mini Kit (Qiagen) according to the manufacturer's protocol, and subsequently transcribed by Transcriptor One-Step RT-PCR Kit (Roche) using MARV Guinea-specific primers. The resulting cDNA was sequenced by Microsynth Seqlab (Sanger sequencing).

Passaging of rescued recMARV Guinea and recMARV Musoke was continued on HuH7 cells until the third passage and on Vero E6 cells until the fifth passage. For recMARV Guinea and recMARV Musoke stock production, fresh Vero E6 cells were infected with 1 ml supernatant of the third passage on HuH7 cells and incubated for 7 dpi at 37 °C. Viral titers (PFU/ml) were calculated based on immunoplaque assays (section 2.2).

Illumina next generation sequencing (NGS) of recMARV Guinea stock virus revealed three mutations A2837R (A or G), A4266T, and G5814GA in non-coding regions. Libraries for next generation sequencing were prepared using the Twist Total Nucleic Acids Library Preparation EF Kit 2.0 (Twist Bioscience) for Viral Pathogen Detection and Characterization, as well as the Twist Target Enrichment Protocol (Rev. 2.0) with the Comprehensive Viral Research Panel. Libraries were sequenced on an Illumina iSeq 100 system, paired end reads 2 x 151 bp. Quality- and adapter trimming of raw reads, mapping and consensus sequence generation were performed using Geneious Prime 2022 build-in tools (BBduk, Geneious mapper, consensus sequence generation highest quality with cutoff 60%, respectively). Detected variants were visually validated in the alignment view. In order to sequence the genome ends, adaptors were ligated to both genome ends using NEBNext® Multiplex Small RNA Library Prep Set for Illumina® kit (NEB) according to the manufacturer's protocol, followed by a reverse transcription PCR using the manufacturer's and recMARV Guinea-specific primers. Afterwards, PCRs using Q5® High-Fidelity DNA Polymerase (NEB) with adaptor- and genome-specific primers were used to generate double-stranded DNA sequences of both genome ends, which were sequenced by Microsynth Seqlab (Sanger sequencing). Detailed sequencing strategy and primers are available on request.

RecMARV Guinea sequence is available under (GenBank accession number OQ847644).

### 2.6 Growth kinetics

HuH7 cells (1x10^6^ cells/T75 flask) were infected with recMARV Guinea, recMARV Musoke, and isolates of MARV Musoke and MARV Leiden using a multiplicity of infection (MOI) of 0.01. Virus-containing inoculum was discarded. Likewise, Vero E6 cells and RoNi/7.1 cells (each 1x10^6^ cells/T75 flask) were infected with recMARV Guinea in comparison to recMARV Musoke with a MOI of 0.01. Growth kinetics in all cell lines were performed in parallel starting with the same virus dilution for each infection. Three biological replicates were performed. Development of CPE was daily monitored and viral titers in the supernatant were determined by 50% tissue culture infectious dose (TCID_50_) assays using Vero E6 cells [26]. Titers were calculated as TCID_50_/ml using the Spearman-Kärber method [27]. Two-way ANOVA with Tukey's multiple comparison tests were performed for statistical analysis with GraphPad Prism (version 8.0.1).

### 2.7 Remdesivir treatment

HuH7 cells (2x10^5^ cells/6 well) were infected with recMARV Guinea and recMARV Musoke using a MOI of 0.1. After removal of the virus-containing inoculum, cells were incubated for 48 h at 37 °C in 3% DMEM ++ containing either 0.1 μM, or 1 μM remdesivir (GS-5734, Biomol)/DMSO or DMSO (0.1%) as control, as well as an untreated control. CPE was monitored after 48 h and viral titers in the supernatants were examined via TCID_50_ assay in Vero E6 cells [26]. Titers were calculated as TCID_50_/ml using the Spearman-Kärber method [27]. Two-way ANOVA with Tukey's multiple comparison test was performed for statistics with GraphPad Prism (version 8.0.1).

### 2.8 Western blot analysis

HuH7 cells were infected with recMARV Guinea, recMARV Musoke and isolates of MARV Musoke and Leiden with a MOI of 0.1. In parallel, HuH7 cells (2x10^5^ cells/6 well) were transfected (*Trans*-IT, Mirrus) with 1 μg plasmid encoding the viral proteins GP, NP, VP40, or VP30 of either MARV Guinea, Musoke [24,25], or Leiden. 48 h later, cells were lysed in sodium dodecyl sulfate (SDS) sample buffer (25% glycerol, 2.5% SDS, 125 mM Tris pH 6.8, 125 mM dithiothreitol, 0.25% bromophenol blue) and boiled for 10 min at 99 °C. Proteins were separated using 10% SDS polyacrylamide gels and transferred onto polyvinylidene difluoride membranes. Membranes were blocked in 10% non-fat dry milk in PBS_def_ for 1 h at room temperature (RT). Staining with the laboratory's own monoclonal primary antibodies (mouse α-GP clone 50-6-10, dilution 1:1000; mouse α-NP clone 59-9-10, dilution 1:1000; mouse α-VP40 clone 40-2-2, dilution 1:1000; mouse α-VP30 clone 11-6-11, dilution 1:1000), that are specific to MARV Musoke, in PBS_def_ supplemented with 1% non-fat dry milk and 0.1% Tween® 20 was performed overnight at 4 °C. Western blot detection was performed with a horseradish peroxidase-conjugated secondary antibody (goat α-mouse HRP, 1:40,000, Dako) using the SuperSignal® West Femto Maximum Substrate (Thermo Scientific), or Luminata Forte Western HRP Substrate (Merck) and ChemiDoc (BioRad).

### 2.9 Indirect immunofluorescence analysis

HuH7 cells (2x10^5^ cells/6 well, seeded on cover slips) were infected with recMARV Guinea, recMARV Musoke and isolates of MARV Musoke, and Leiden (MOI of 0.1). 24 hpi cells were fixed with 4% PFA/DMEM for 48 h. After washing with PBS_def_, cells were permeabilized with 0.5% Triton X-100 in PBS_def_ for 10 min, treated with 0.1 M glycine for 10 min, and afterwards incubated in blocking buffer (containing 2% bovine serum albumin; 5% Glycerin; 0.2% Tween® 20; 0.05% NaN3 in PBS_def_) for 10 min. Primary antibodies (monoclonal mouse α-VP40 clone 40-2-2, dilution 1:50; polyclonal chicken α-NP 102, dilution 1:100) were diluted in blocking buffer. Finally, staining with fluorescence-labeled secondary antibodies (goat α-chicken IgY (H + L) AlexaFluor™ 594, dilution 1:400, Invitrogen; goat α-mouse IgG (H + L) Cross-Adsorbed AlexaFluor™ 488, dilution 1:400, Invitrogen) followed. DAPI (4′,6-diamidino-2′-phenylindol) was added at a dilution of 1:2000.

### 2.10 Electron microscopy

HuH7 cells (60% confluence/T75 flask) were infected with recMARV Guinea (MOI of 0.15) and recMARV Musoke (MOI of 0.2), respectively. Viral particles in the supernatants were concentrated via ultracentrifugation (25,000 rpm for 90 min at 4 °C) at 3 dpi. Fixation of virions was performed using 4% PFA/DMEM for 48 h. Fixed viral particles were attached to formvar-coated TEM grids and negatively stained with 1% phosphotungstic acid. Electron micrographs were recorded at 80 kV on a JEOL JEM-1400 transmission electron microscope (JEOL, Tokyo, Japan) using a TemCam-F416 camera (TVIPS). Three independent virion preparations of each virus were analyzed, and the length of at least 50 virions per preparation was measured with the help of analytical tools in ImageJ [28].

## 3 Results

### 3.1 Functional validation of complemented MARV Guinea genome ends using a MARV Guinea-specific minigenome assay

**Fig. 1:**
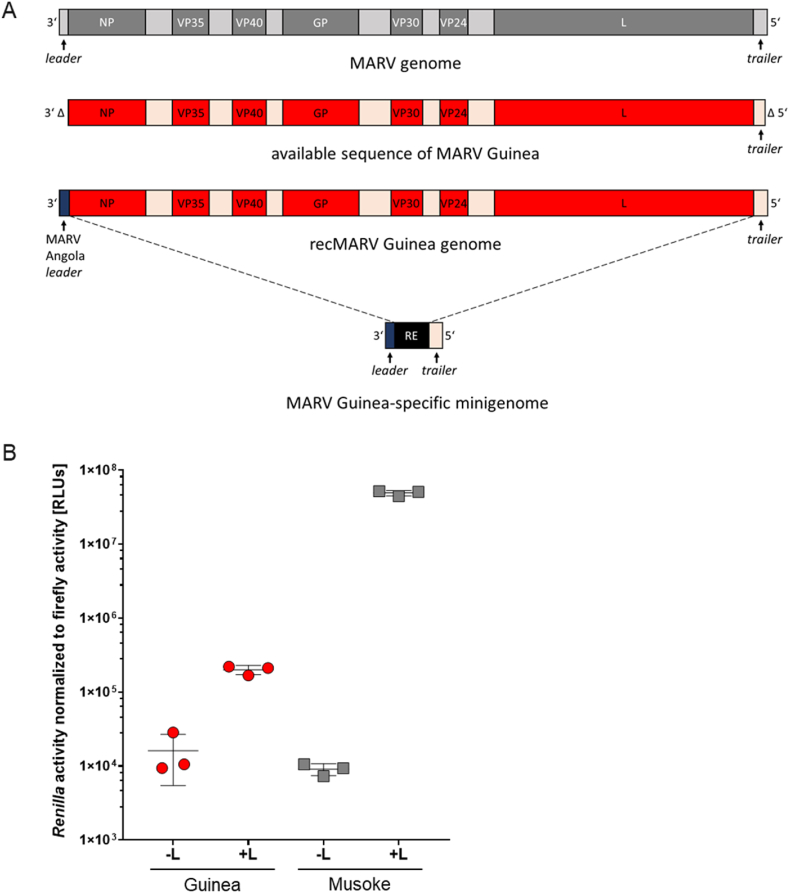
(A) Overview of the MARV genome with open reading frames of the viral proteins (dark grey), untranslated 3′ *leader*, 5′ *trailer,* and intergenic regions (light grey) (above). Originally available sequence of MARV Guinea [14] (middle; Δ: missing nts in the original sequence) and recMARV Guinea sequence complemented with MARV Angola nts (below). MARV Guinea-specific monocistronic minigenome containing the reconstituted genome ends (below). (B) MARV-specific minigenome reporter gene assay. HuH7 cells were transfected with plasmids encoding the MARV Guinea-specific nucleocapsid proteins L, NP, VP35, and VP30 (red). Additionally, plasmids encoding the MARV Guinea-specific minigenome under the control of a T7 promotor, a T7 polymerase, and a Firefly luciferase were transfected. As a negative control viral polymerase L was omitted (-L). As a positive control, the same setup was used with MARV Musoke-specific nucleocapsid proteins and minigenome (grey). 48 hpt, cells were lysed and *Renilla* reporter gene activity was measured. Firefly luciferase activity was measured for normalization. Shown is the mean of three independent experiments in *Renilla* light units (RLU), SD is indicated by error bars.

Since the original sequence of MARV Guinea (GenBank accession number OK665848) did not contain the complete genome ends (sequence lacked the first 119 nts of the *leader*, the first 16 nts of the *NP* gene and the last 10 nts of the *trailer* region), we substituted the missing nts with respective nts of a closely related MARV Angola sequence (GenBank accession number DQ447654) (Fig. 1A). The introduced *leader* sequence is generally highly conserved among all MARV strains. To validate the functionality of the complemented MARV Guinea genome ends as well as the functionality of the MARV Guinea nucleocapsid proteins L, NP, VP35, and VP30, we first established a MARV Guinea-specific minigenome reporter assay containing a *Renilla* luciferase as reporter gene (Fig. 1A) [24,25]. After transfection, this T7-driven minigenome is transcribed and replicated by the viral nucleocapsid proteins, transcription activation is detected upon *Renilla* luciferase activity. Reporter gene activity was increased by 1.5 log-fold in HuH7 cells when compared to cells transfected without the viral polymerase L (-L) (Fig. 1B, red bars). This indicates MARV Guinea-specific activation of viral transcription and replication and suggests both, a functional MARV Guinea minigenome and functional MARV Guinea nucleocapsid proteins. Interestingly, when comparing the MARV Guinea minigenome system with the well-established MARV Musoke minigenome system (Fig. 1B, grey bars), luciferase levels were significantly lower for MARV Guinea. We also assessed reporter gene activity of the MARV Guinea minigenome system in HEK293F cells, but could not detect any specific reporter gene signal (data not shown). This could suggest that adjustments in transfected DNA amounts and/or ratios between viral proteins are required to improve and optimize the MARV Guinea minigenome system as it has been shown during the establishment of the MARV Musoke minigenome system where especially the ratio between NP and VP35 was crucial for efficient minigenome transcription.

[25]. We also cannot rule out an impairment of MARV Guinea reporter gene activity due to reconstruction of the missing genome ends by sequences from MARV Angola, although this is unlikely because, in particular, the leader sequences are 98–100% conserved between the different MARV strains (Musoke, Leiden, and different virus isolates from Angola), only 0–2 nts differ from a total of 103 nts (Geneious sequence alignment not shown). Taken together, we concluded that the complemented genome ends, as well as the viral nucleocapsid proteins of MARV Guinea are basically functional in HuH7 cells.

### 3.2 Rescue of recombinant MARV Guinea

**Fig. 2:**
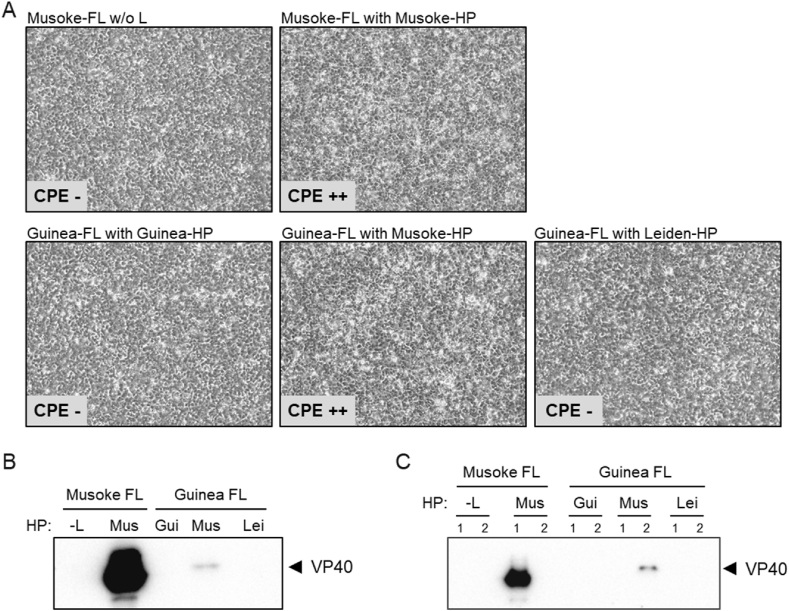
(A) Development of cytopathic effect (CPE) in Vero E6 cells, passage 3 at 7 dpi. FL: full-length plasmid; HP: helper plasmids NP, VP35, VP30, L., w/o: without (B, C) Supernatants were analyzed by western blotting for the presence of VP40 with a monoclonal antibody detecting both MARV Musoke and Guinea VP40. -L: without polymerase L; Mus: Musoke; Gui: Guinea; Lei: Leiden. (B) Vero E6 cells, passage 2, 7 dpi (C) HuH7 cells, passage 2, 7 dpi. Technical duplicates are indicated by numbers. For uncropped images of B) and C) see Supplement Fig. S1.

After having verified the functionality of the *leader* and *trailer* regions of the MARV Guinea genome and the MARV Guinea nucleocapsid proteins L, NP, VP35, and VP30, we cloned a full-length (FL) MARV Guinea plasmid in order to generate recMARV Guinea (Fig. 1A). We started virus rescue experiments on a mixture of Vero E6 cells and HuH7 cells [21]. In parallel to the recMARV Guinea setting, we included an experimental setup with helper plasmids of MARV Musoke, or MARV Leiden, respectively (Table 1). We also included a FL recMARV Musoke plasmid together with homologous helper plasmids as positive control. Serial passaging was performed on both, Vero E6 or HuH7 cells, respectively. Interestingly, after three passages a pronounced CPE developed in Vero E6 cells for recMARV Guinea when using heterologous helper plasmids from MARV Musoke (Fig. 2A). Rescue of recMARV Guinea was neither successful with homologous MARV Guinea helper plasmids, nor with heterologous MARV Leiden helper plasmids (Table 1) despite the fact that the homologous MARV Guinea proteins are expressed and functional in our reporter gene assay (Fig. 1B). Successful rescue of recMARV Guinea was confirmed by cDNA sequencing of viral RNA and presence of the viral matrix protein VP40 in the supernatant by Western blot analysis (Fig. 2B and C). NGS sequencing of stock virus confirmed the successful rescue of recMARV Guinea, yet mutations were detected in non-coding regions of recMARV Guinea when compared with the original FL cDNA sequence (A2837R, A4266T, G5814GA). Sequences of recMARV Guinea are available under GenBank accession number OQ847644.

**Table 1:** Rescue settings with different full-length plasmid and helper plasmid combinations.

recMARV	Full-length plasmid	Helper plasmids	Successful rescue
negative control	Musoke	Musoke w/o L	no
recMARV Musoke	Musoke	Musoke	yes
recMARV Guinea	Guinea	Guinea	no
recMARV Guinea	Guinea	Musoke	yes
recMARV Guinea	Guinea	Leiden	no

### 3.3 Detection of recMARV Guinea proteins by MARV Musoke-specific antibodies

**Fig. 3:**
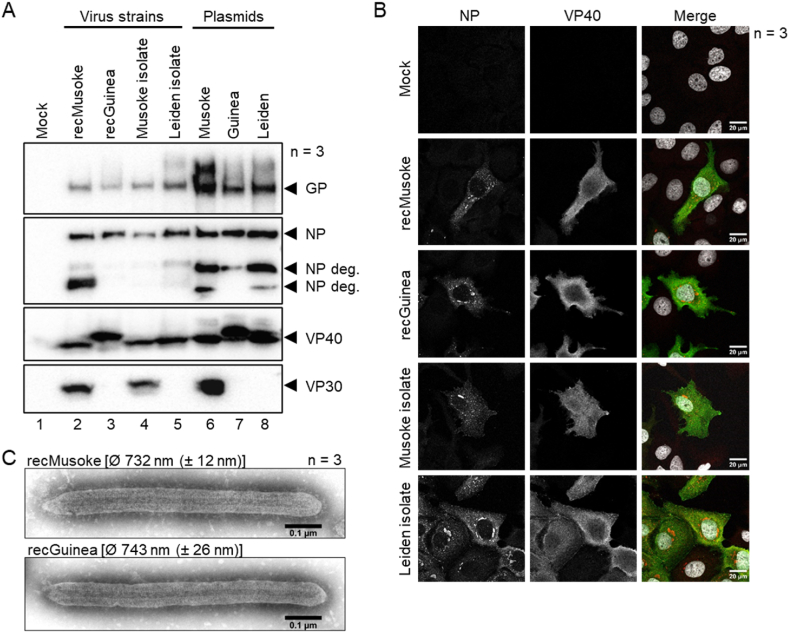
(A) HuH7 cells were infected with recMARV Guinea, recMARV Musoke, and isolates of MARV Musoke and Leiden. In parallel, HuH7 cells were transfected with plasmids encoding viral proteins GP, NP, VP40 or VP30 of either MARV Guinea, Musoke, or Leiden. 48 h later, cell lysates were submitted to Western blot analysis using monoclonal antibodies specific for MARV Musoke GP, NP, VP40, or VP30. Three independent experiments were performed. Deg.: degradation band. For uncropped images see supplement Fig. S2. (B) HuH7 cells were infected with recMARV Guinea, recMARV Musoke, and isolates of MARV Musoke and Leiden. 24 hpi cells were fixed with 4% PFA and immunofluorescence analysis was performed using NP- (red), and VP40-(green) specific antibodies. Nuclei were stained using DAPI (grey). Three independent experiments were performed. (C) For electron microscopy, HuH7 cells were infected with recMARV Guinea and recMARV Musoke. Supernatants were purified by ultracentrifugation 3 dpi. Negative staining was performed using 1% phosphotungstic acid. Three independent experiments were performed. For uncropped images see Supplement Fig. S3.

We next wanted to assess whether our MARV Musoke-specific antibodies would also detect MARV Guinea viral proteins. We therefore performed Western blot analysis of cell lysates of HuH7 cells infected with recMARV Guinea, recMARV Musoke, as well as isolates of MARV Musoke and Leiden (MOI of 0.1, 48 hpi). In parallel, we transfected HuH7 cells with plasmids coding for GP, NP, VP40, or VP30 of either MARV Guinea, Musoke, or Leiden. Monoclonal antibodies (mAB) against GP, NP, and VP40 were able to detect MARV Guinea, as well as MARV Musoke and Leiden viral proteins (Fig. 3A). Interestingly, MARV Guinea VP40 expression showed a distinct size shift in the gel in both, infected and transfected cells (Fig. 3A, lanes 3 and 7). This suggests different post-translational modifications of MARV Guinea in comparison to VP40 of MARV Musoke and Leiden, since the overall number of amino acids (aa) as well as the calculated molecular weight of VP40 is equal (both 303 aa, molecular weight 33.79 kDa). A MARV Musoke VP30-specific mAB was not able to detect VP30 from MARV Guinea, nor Leiden (Fig. 3A, lanes 3 and 7, lanes 5 and 8, respectively). Immunofluorescence analyses of infected HuH7 cells showed no differences in cellular localization of NP and VP40 among different MARV strains. While NP showed a clear concentration in viral inclusion bodies, VP40 was diffusely distributed in the cytoplasm of the cell (Fig. 3B) [29,30].

Morphological characterization of recMARV Guinea and recMARV Musoke virions using negative staining electron microscopy revealed no obvious differences, with both viruses exhibiting the filamentous shape typically observed for MARV particles (Fig. 3C). The average length of virions was comparable, with 743 nm (±26 nm) measured for recMARV Guinea and 732 nm (±12 nm) for recMARV Musoke.

### 3.4 *In vitro* characterization of recMARV Guinea in comparison with other MARV isolates

**Fig. 4:**
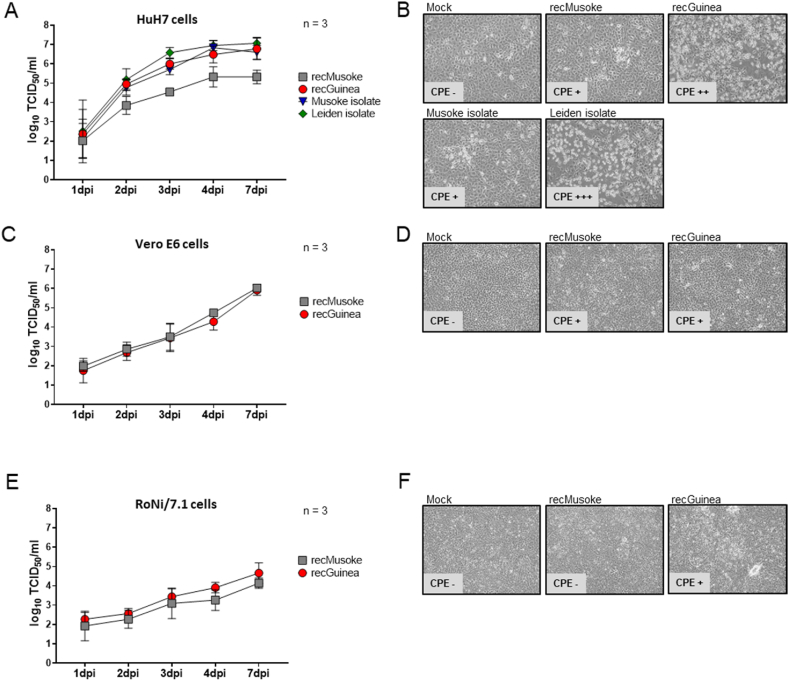
(A) HuH7 cells (human) were infected with recMARV Guinea, recMARV Musoke, and isolates of MARV Musoke and Leiden for replication kinetics. Viral titers from supernatants calculated as TCID_50_/ml. Three independent experiments were performed, SD is indicated by error bars. (B) Cytopathic effect (CPE) in HuH7 cells at 3 dpi (C) Vero E6 cells (African green monkey) were infected with recMARV Guinea and recMARV Musoke. Viral titers from supernatants were analyzed as described in (A). (D) CPE in Vero E6 cells at day 7. (E) RoNi/7.1 cells (fruit bat) were infected with recMARV Guinea and recMARV Musoke. Viral titers from supernatants were analyzed as described in (A). (F) CPE in RoNi/7.1 cells at day 7.

To characterize the propagation of recMARV Guinea, we performed comparative growth kinetics (MOI of 0.01) with different MARV strains in relevant human (HuH7), monkey (Vero E6), and bat (RoNi/7.1) cell lines. In HuH7 cells, recMARV Guinea replicated to similar viral titers as the natural isolates of MARV Musoke and Leiden. In contrast to that, replication of recMARV Musoke was significantly (p-value <0.05) attenuated up to 2 log-scales until day 7 (Fig. 4A). Interestingly, development of CPE was more pronounced in HuH7 cells infected with recMARV Guinea, comparable with MARV Leiden, while CPE for both, recMARV Musoke and MARV Musoke isolate was barely visible (Fig. 4B). When we compared the growth of recMARV Guinea with recMARV Musoke in Vero E6 cells (MOI of 0.01), both viruses replicated to similar titers (Fig. 4C), in contrast to HuH7 cells (Fig. 4A). CPE in recMARV Guinea-infected cells was comparable to cells infected with recMARV Musoke (Fig. 4D), which was different to HuH7 cells (Fig. 4B) We also used fruit bat kidney cells from the MARV reservoir *Rousettus aegyptiacus* (RoNi/7.1) for growth kinetics. Interestingly, while at earlier time points, recMARV Guinea and recMARV Musoke replicated to similar titers, at day 4 and 7 post infection recMARV Guinea showed a replicative advantage over recMARV Musoke (Fig. 4E). CPE in bat cells was not detected for recMARV Musoke and overall weaker for recMARV Guinea when compared with HuH7 cells (Fig. 4F). Overall, when comparing the viral end titers, it became clear that titers were highest in human HuH7 cells (for all tested MARV strains), following monkey Vero E6 cells and bat RoNi/7.1 cells indicating that the investigated MARV strains replicated most efficiently in the human cell line.

### 3.5 Remdesivir strongly reduces replication of recMARV Guinea

**Fig. 5:**
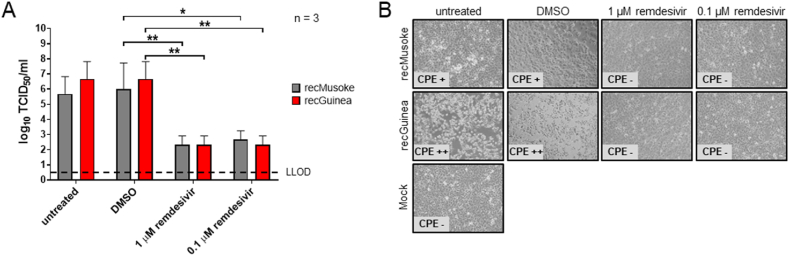
(A) HuH7 cells were infected with recMARV Guinea and recMARV Musoke. Cells were incubated for 48 h in 3% DMEM ++ (untreated control), or in 3% DMEM ++ containing either DMSO, 1 μM, or 0.1 μM remdesivir. Viral titers from supernatants were calculated as TCID_50_/ml. Three independent experiments were performed, SD is indicated by error bars. LLOD, lower limit of detection, stars indicate statistical significance (* p-value ≤0.05; ** p-value ≤0.01). (B) Cytopathic effect (CPE) was monitored 48 hpi.

Remdesivir is a nucleoside analogue that acts as a potent inhibitor of viral RNA-dependent RNA polymerases with a broad-spectrum activity against a variety of different viruses including coronaviruses or paramyxoviruses [[31], [32], [33]]. The antiviral activity of remdesivir against filoviruses has been previously reported in both, *in vitro* and *in vivo* models for EBOV and MARV [[34], [35], [36], [37]]. To analyze whether remdesivir also inhibits replication of MARV Guinea *in vitro*, we infected HuH7 cells with either recMARV Guinea or recMARV Musoke followed by a treatment with different concentrations of remdesivir (1 μM versus 0.1 μM). As expected, viral titers of recMARV Guinea as well as recMARV Musoke were significantly reduced upon treatment with remdesivir at both concentrations compared to DMSO-treated cells (Fig. 5A), which was in line with the reduced development of CPE (Fig. 5B).

## 4 Discussion

The relevance of MARV as a causative public health pathogen is underlined by increasing sporadic MARV outbreaks in the last years [17]. This includes emerging of the virus in the sub-Saharan Africa region like recent MARV outbreaks in Equatorial Guinea [38], already being the third largest MARV outbreak to date [13], as well as in Tanzania [39], but also first-time MARV outbreaks in West Africa: 2021 in Guinea and 2022 in Ghana. Fortunately, the West African outbreaks were limited to only a few fatal cases [13]. However, the potential of sporadic MARV outbreaks in West Africa is nevertheless present, since MARV-positive *Rousettus aegyptiacus* fruit bats, the reservoir of MARV, have been first-ever identified in the Gueckedou prefecture in Guinea, increasing the possibility of further spill-over events [7].

Since there exists no MARV isolate from that West African outbreak in Guinea [14], we used a reverse genetic approach to generate a recombinant MARV based on available sequences [14], which allowed the characterization of viral replication in relevant cell lines and the evaluation of therapeutic approaches. In our reverse genetic approach, we replaced both missing genome ends as well as internal nts with sequences of a closely related MARV Angola sequence (GenBank accession number DQ447654; amino acid identity NP 98.705%, VP40 99.01%, VP35 99.696%, GP 97.504%, VP30 100%, VP24 99.605%, L 99.056%). The substituted genome ends as well as the viral nucleocapsid proteins NP, VP35, VP30, and L were proven to be functional in a MARV Guinea-specific minigenome assay in HuH7 cells. However, compared to the well-established reference minigenome system based on MARV Musoke, luciferase levels obtained with the MARV Guinea-specific minigenome assay were significantly lower in HuH7 cells and lacked any specific signal in HEK293F cells. This suggests that further improvement of the assay system is needed by titrating the amounts of viral proteins and their ratios, as has already been shown in the development of the MARV-Musoke minigenome system [25]. Interestingly, infectious recMARV Guinea could be rescued with helper proteins NP, L, VP35, and VP30 of MARV Musoke, but not with homologous MARV Guinea proteins. Since the reporter gene activity of the MARV Guinea-specific minigenome was also less efficient compared to the MARV Musoke minigenome system, this might again suggest inefficient initial steps of viral RNA synthesis, likely because of suboptimal ratios between viral proteins. Because the rescued and replicating recMARV Guinea did not show any attenuation in terms of virus propagation, this indicates fully functional MARV Guinea proteins. In addition, Western blot analysis revealed similar expression levels of proteins of MARV Musoke and Guinea for NP, VP40, and GP (Fig. 3A), as well as for VP35, VP30, and VP24 (data not shown). Again, this rather points in the direction that optimal plasmid conditions for reverse genetic systems might vary between different MARV strains, especially based on the previous data demonstrating the importance of the optimal ratio between NP and VP35 [25], than fundamental problems of the MARV Guinea proteins since the rescued recMARV Guinea did not show any growth defects compared to other MARV strains. The successful rescue of recMARV Guinea as well as the fact that stock viruses did not show substantial sequence changes in the *leader* and *trailer* region suggests at least some flexibility of the MARV polymerase complexes at those regions as it is reflected by rescue of a recMARV Guinea with complemented MARV Angola genome ends by a MARV Musoke-specific polymerase complex. However, we identified three mutations in internal non-coding regions during virus rescue. Whether these mutations affect viral fitness and/or reflect compensatory mutations in terms of cell culture adaptation, is so far not understood.

As could be expected, particle appearance and overall length was comparable for recMARV Guinea vs. recMARV Musoke. Western blot analyses with available monoclonal antibodies against MARV Musoke detected also MARV Guinea proteins NP, GP, and VP40. However, a monoclonal antibody for VP30 was not able to detect neither VP30 from MARV Guinea, nor from MARV Leiden suggesting that the epitope is missing here. Despite having a similar theoretical molecular weight, VP40 from MARV Guinea migrated significantly higher than VP40 from MARV Musoke or Leiden in a SDS-PAGE. Comparing the amino acid sequences of recMARV Guinea with MARV Musoke and Leiden (both 303 aa in total), revealed only two amino acid changes, M44V and R56N. Whether these amino acid changes contribute to an altered or modified post-translational modification is currently under investigation.

The subsequent characterization of growth kinetics revealed that recMARV Guinea replicated most efficiently in human HuH7 cells. The natural isolates MARV Musoke and Leiden showed comparable growth rates, however, a recMARV Musoke replicated to lower titers, for so far unknown reasons. Interestingly, infection of HuH7 cells with recMARV Guinea leads to the development of a dominant CPE, comparable with MARV Leiden, while CPE for both, recMARV Musoke and MARV Musoke isolate was barely visible, potentially due to cell culture adaptation effects of MARV Musoke. The difference in replication rates between both recombinant viruses - recMARV Guinea vs. recMARV Musoke - was not seen in Vero E6 cells that are type I IFN–deficient cells [40]. In fruit bat cells RoNi/7.1, recMARV Guinea showed viral end titers slightly higher than recMARV Musoke. In contrast to human HuH7 cells, development of CPE in RoNi/7.1 cells was strongly reduced upon infection with recMARV Guinea. Overall titers were highest in human HuH7 cells and lowest in fruit bat cells RoNi/7.1, which might be attributed to a better established antiviral response in bat cells [41]. In a transcriptome study, a direct comparison of HuH7 cells and embryonal *Rousettus aegyptiacus* cells could show that the expression of *toll-like receptor 3* gene was increased in HuH7 cells after MARV Musoke infection, indicating an activation of a type-I interferon response, which could cause a more pronounced CPE in HuH7 cells [41]. This was not the case in the *Rousettus* cells, which may explain the only faintly visible CPE of recMARV Guinea and the missing CPE of recMARV Musoke in the RoNi/7.1 cells.

The adenosine analogue remdesivir was initially developed for the treatment of EBOV disease and was already published to reduce replication of MARV Angola in macaques and against different MARV strains in HeLa cells [34,37,42]. In our study, remdesivir proved to efficiently reduce viral titers of both recMARV Guinea and recMARV Musoke in HuH7 cells at a concentration of 0.1 and 1 μM, respectively.

In conclusion, we were able to successfully rescue recMARV Guinea and initial results show effective replication in various cell lines, including human hepatoma cells. Furthermore, inhibition of viral replication in cell culture by remdesivir could be demonstrated, underlining its potential therapeutic option for future MARV outbreaks. The generation of recombinant viruses such as recMARV Guinea not only enables characterization of viral replication kinetics of a new emerging virus, when virus isolates are missing, but furthermore allows the evaluation of potential antivirals as well as vaccine efficacy studies to combat potential future outbreaks of closely related MARV strains that are endemic in Africa.

## Author contribution statement

Isabel von Creytz: Conceived and designed the experiments; Performed the experiments; Analyzed and interpreted the data; Wrote the paper.

Gesche K Gerresheim; Martin Schauflinger; Lennart Kämper: Conceived and designed the experiments; Performed the experiments.

Clemens Lier: Performed the experiments, Analyzed and interpreted the data.

Jana Schneider; Marcel Benz: Performed the experiments.

Cornelius Rohde: Contributed reagents, material and analysis data-

Markus Eickmann: Conceived and designed the experiments.

Nadine Biedenkopf: Conceived and designed the experiments; Analyzed and interpreted the data; Wrote the paper.

## Data availability statement

Data associated with this study has been deposited at NCBI GenBank with the accession number OQ847644.

## Declaration of competing interest

The authors declare that they have no known competing financial interests or personal relationships that could have appeared to influence the work reported in this paper.
