# Supplementary figures and images for: Rescue and characterization of the first West African Marburg virus 2021 from Guinea
Source: Heliyon. 2023 Aug 29;9(9):e19613. doi: 10.1016/j.heliyon.2023.e19613 (PMC10558868; doi:10.1016/j.heliyon.2023.e19613)

**Figure S1**  
Uncropped image of Figure 2B

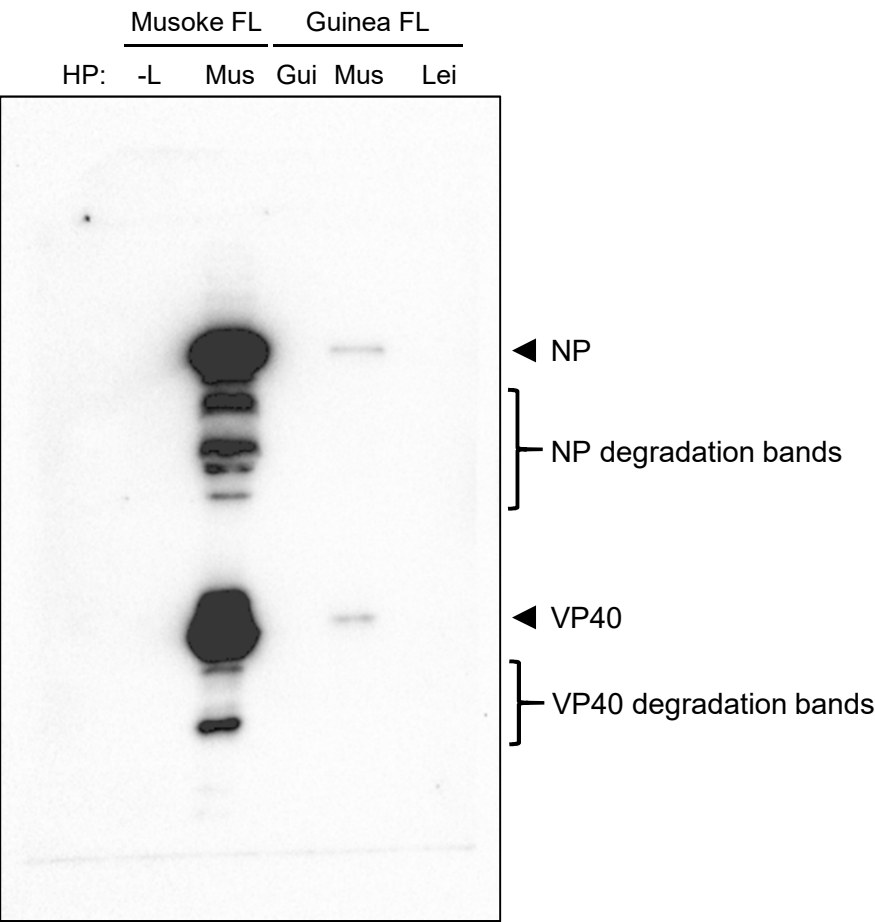

Uncropped image of Figure 2C

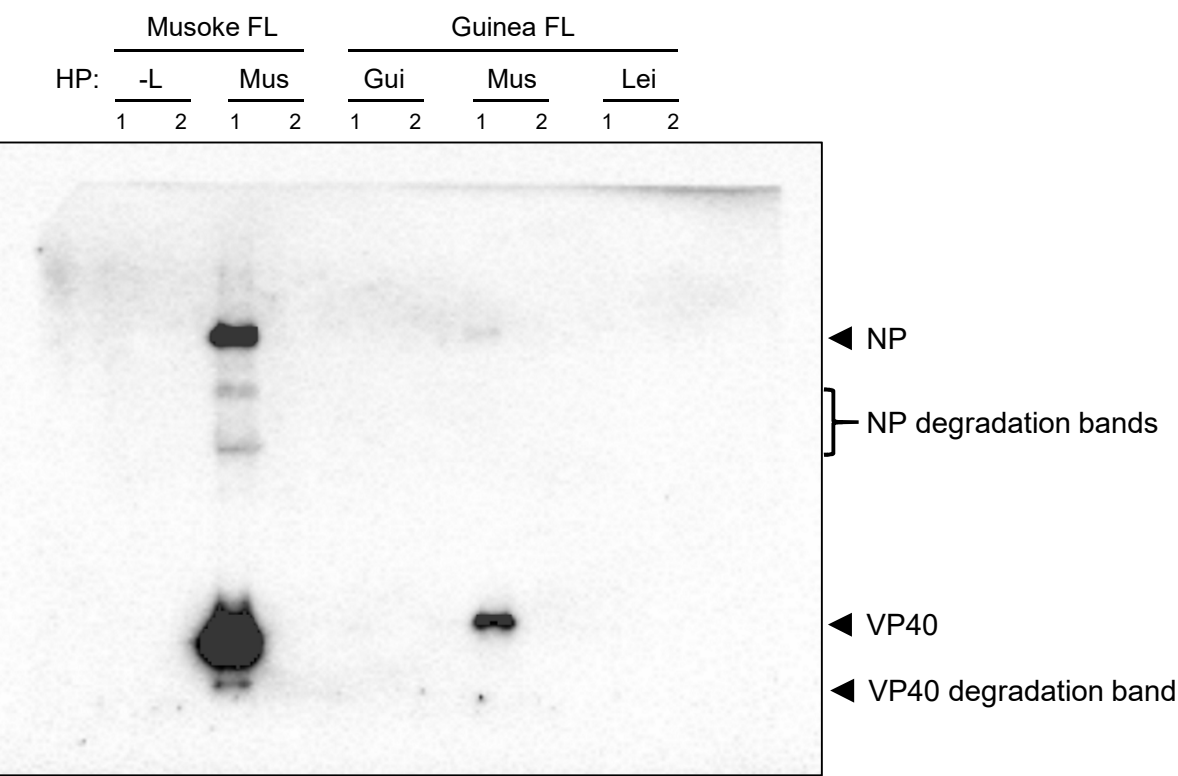

Supplement: Multimedia component 1 [file mmc1.pdf]

Figure S2

Uncropped image of Figure 3A

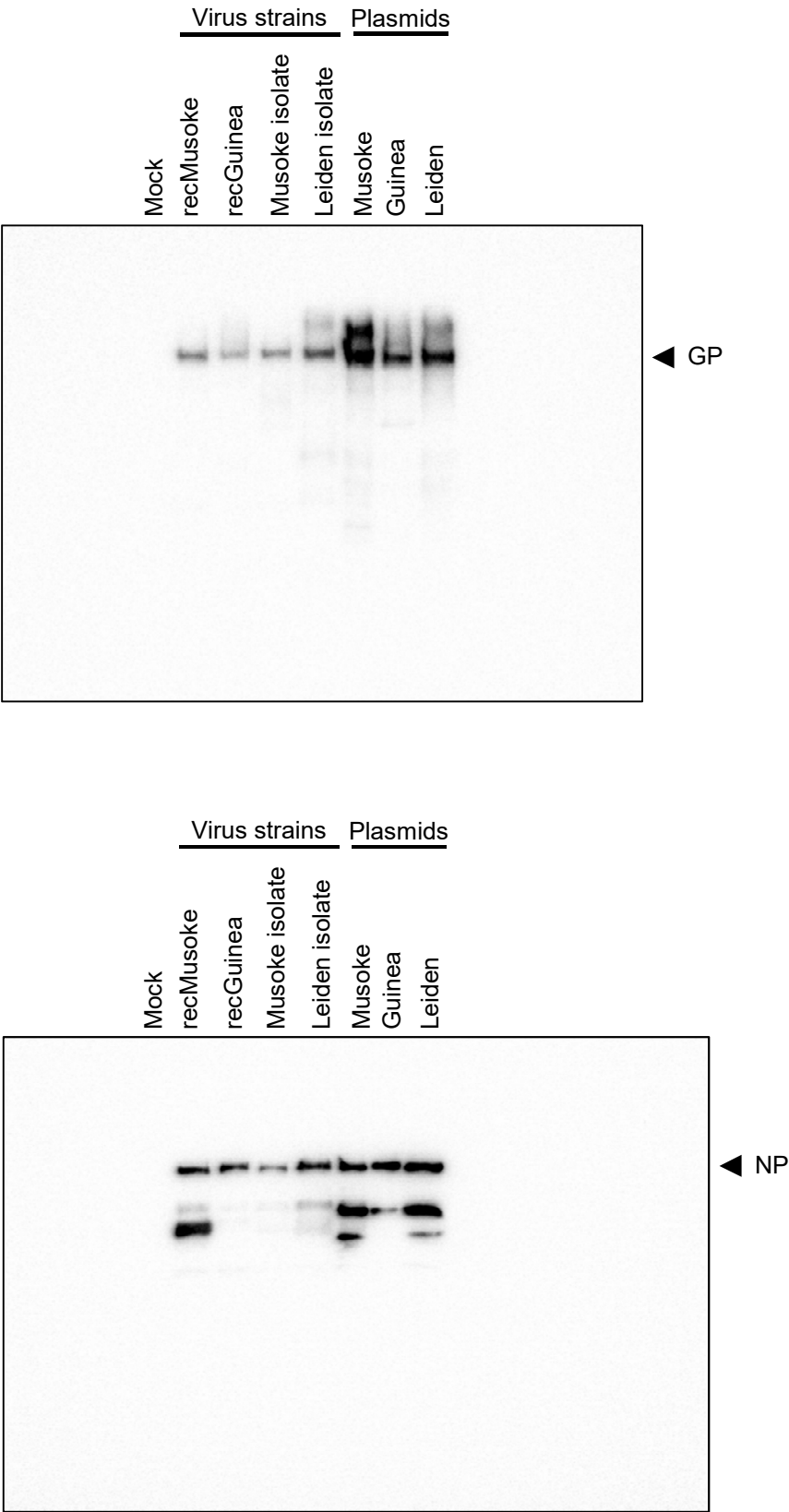

Figure S2

Uncropped image of Figure 3A

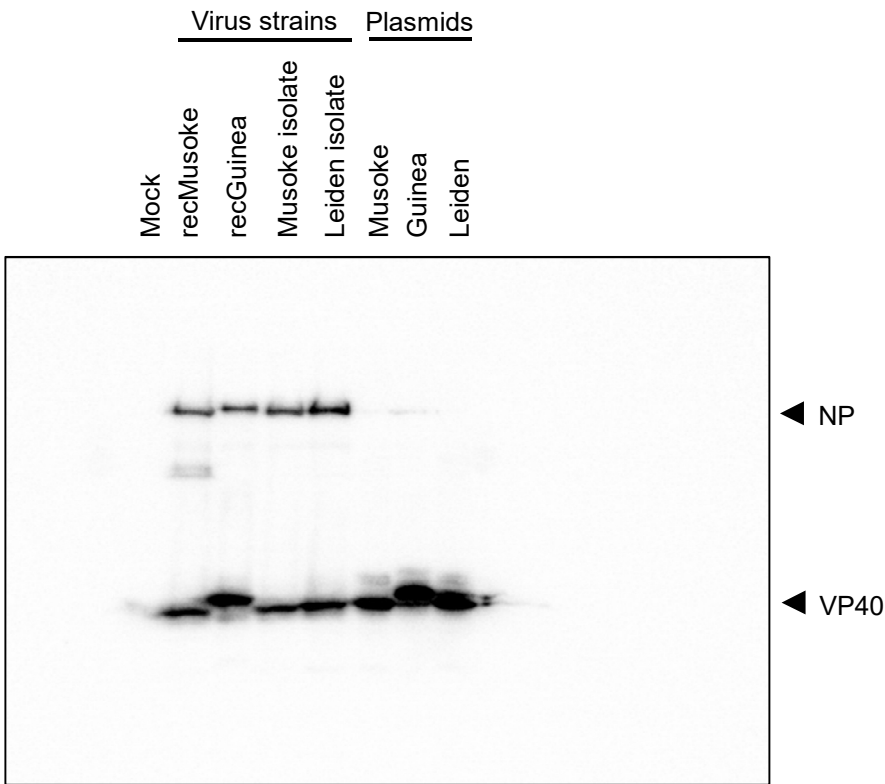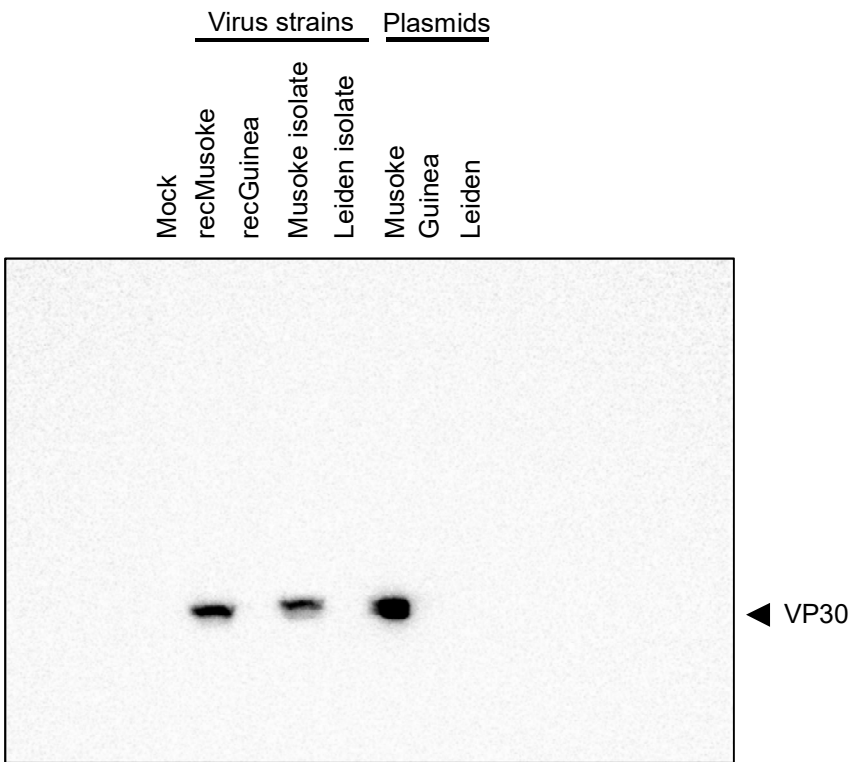

Supplement: Multimedia component 2 [file mmc2.pdf]

## Figure S3

Uncropped image of Figure 3C

recMARV Musoke

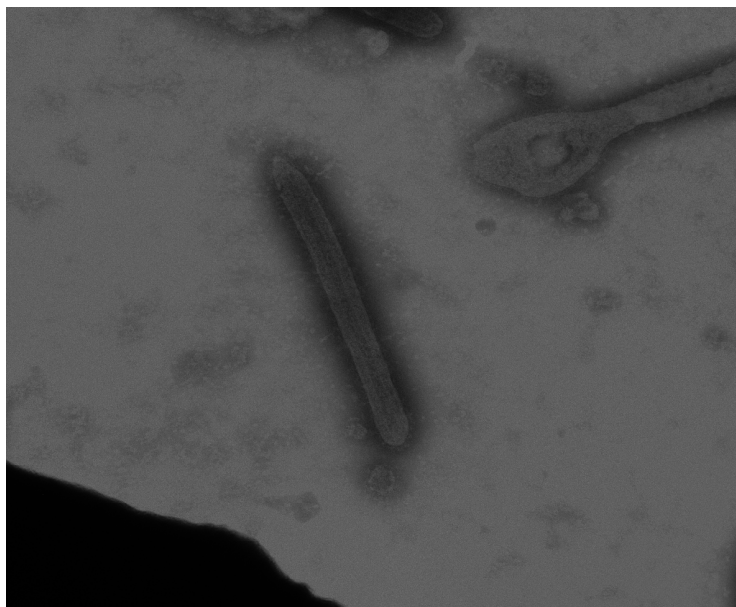

recMARV Guinea

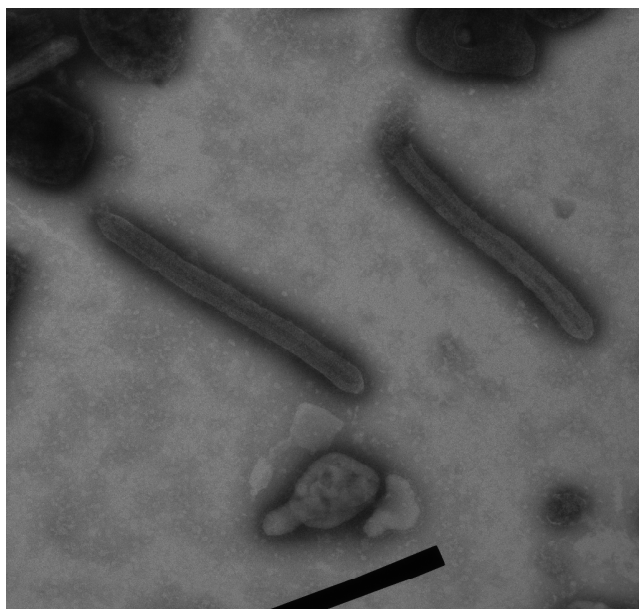

Supplement: Multimedia component 3 [file mmc3.pdf]
